# *Tinea corporis* caused by *Trichophyton benhamiae*: report of the first case transmitted by guinea pig in Brazil^☆^

**DOI:** 10.1016/j.abd.2023.06.011

**Published:** 2024-02-24

**Authors:** Cristiana Ludwig Schneider Longo, Flávio Marcondes Hercules, Fábio Silva de Azevedo, Adriana Lúcia Pires Ferreira, Rosane Orofino-Costa

**Affiliations:** a Private Practice, Rio de Janeiro, RJ, Brazil; b Laboratórios DASA S/A, Rio de Janeiro, RJ, Brazil; c Discipline of Dermatology, Faculty of Medical Sciences, Universidade do Estado do Rio de Janeiro, Rio de Janeiro, RJ, Brazil

Dear Editor,

*Trichophyton benhamiae (T. benhamiae)* is an emerging zoophilic dermatophyte, an important causative agent of dermatophytosis in several parts of the world. It is transmitted mainly by guinea pigs (*Cavia porcellus*), and its identification is carried out by molecular and proteomic methods, in addition to morphological ones.

We report the first case of *tinea corporis* in a child living in Rio de Janeiro, Brazil, transmitted by a guinea pig.

A ten-year-old, healthy white female patient seeked medical care because two weeks before, she had presented with multiple, mildly pruritic lesions on the neck, submandibular region, left forearm, and right scapular region, ranging from 2‒6 cm in diameter (Fig. 1). She had been in contact with a guinea pig for one month which was initially healthy, but which developed a skin lesion after ten days.

**Figure 1:**
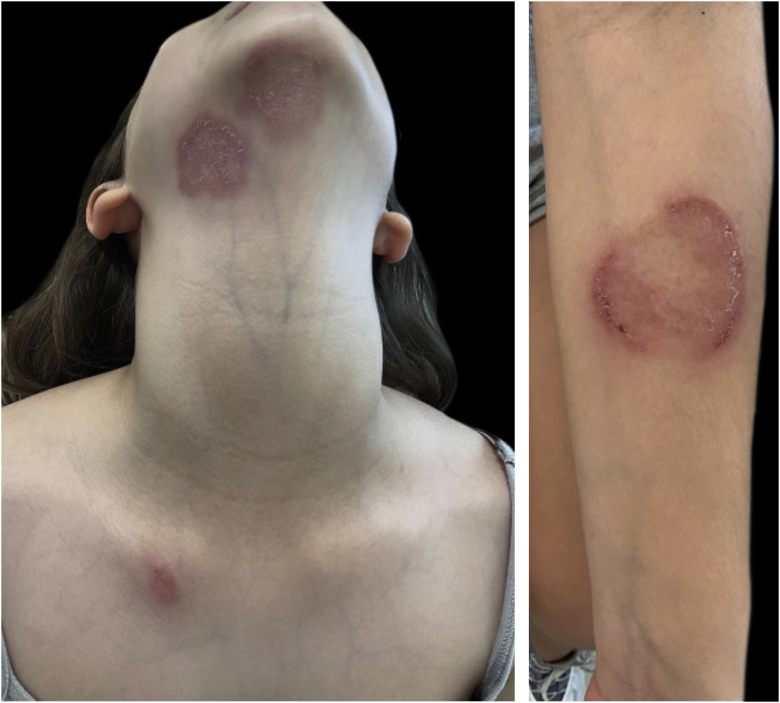
Erythematous-desquamative, circinated, vesico-crusted plaques with well-defined edges. They show centrifugal growth and central clearing.

Direct mycological examination of the skin desquamation showed septate hyaline hyphae and arthroconidia (Fig. 2). *Trichophyton benhamiae* was isolated and identified by its macro- and micromorphological aspects (Fig. 3 A‒D) and by proteomic analysis (MALDI ToF-MS, Biomerieux, confidence level 99.9%).

**Figure 2:**
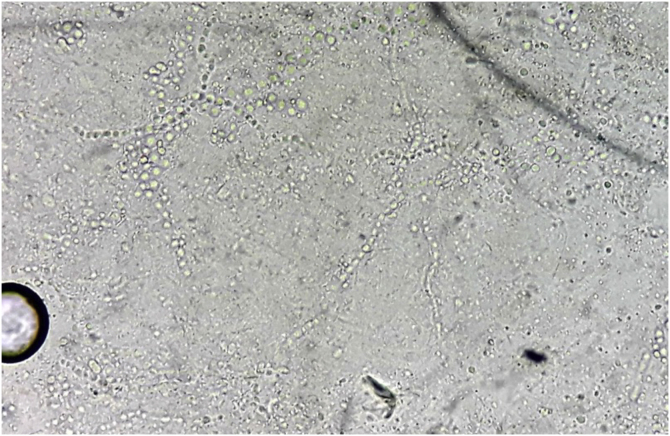
Direct mycological examination: skin desquamation containing septate, branched hyaline hyphae and arthroconidia. KOH 40% (×100).

**Figure 3:**
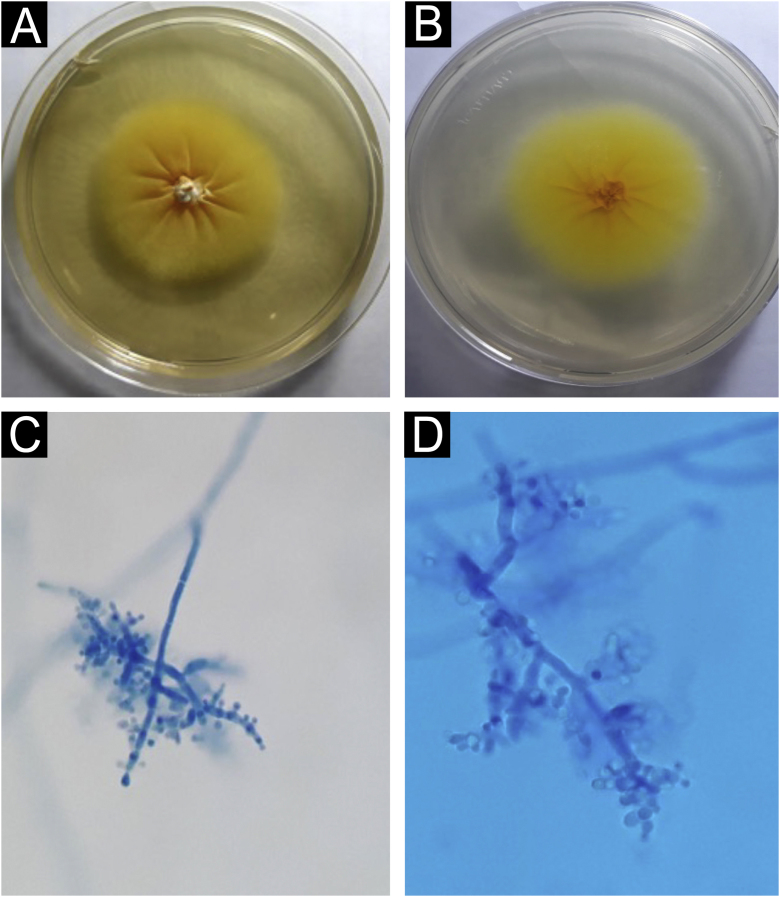
*Trichophyton benhamiae*. (A) Macromorphology of the colony showing a yellowish-beige, velvety surface with radial growth. (B) Back of the colony, bright yellow; (C and D) micromorphology, with septate, branched hyaline hyphae and rounded, pyriform microconidia, grouped in clusters or arranged laterally and at the ends of the hyphae. Lactophenol-cotton blue (×100 and ×400).

She started treatment with oral terbinafine, 250 mg/day, for four weeks, with complete resolution of the lesions (Fig. 4). The animal was treated by the veterinarian with clotrimazole cream, applied twice a day for two weeks, with improvement of the condition.

**Figure 4:**
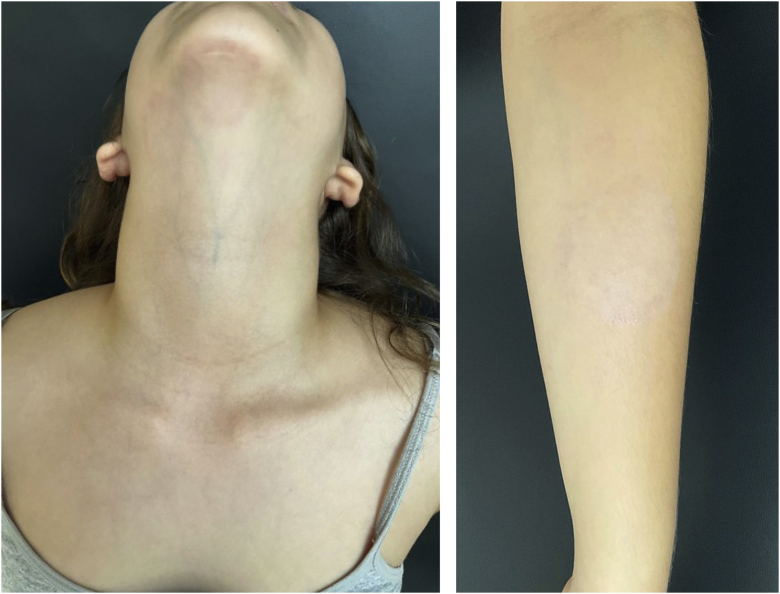
Complete resolution after 30 days of oral treatment.

*Trichophyton benhamiae*, first described by Ajello and Cheng in 1967,^1^ is a zoophilic dermatophyte fungus that has gained global prominence in the last 20 years.^2^

The first case of infection in humans was published in 2002, in Japan.^3^ Since then, cases have been reported in Germany and other European countries, as well as in China and the Americas, and their prevalence has been increasing, especially in Germany, Switzerland, and Japan.^4^ Fratti et al., in a recent Swiss study that analyzed the prevalence of dermatophytes in different animals, showed that *T. benhamiae* was the most prevalent dermatophyte in guinea pigs, found in 48 of 50 animals,^5^ data confirmed in another prevalence study carried out in Germany.^4^

In addition to the guinea pig, transmission can occur through other animals, such as small rodents, rabbits, cats, dogs, and porcupines.^2^, ^6^, ^7^ The first and only case of *tinea corporis* caused by *T. benhamiae*, reported in Brazil, was transmitted by a cat.^8^

Berlin et al. concluded, in a study on the prevalence and dissemination factors of *T. benhamiae*, that guinea pigs can be asymptomatic carriers: 92.7% of the colonized animals did not have any apparent lesions.^4^

Clinically, the lesions have an inflammatory nature, due to the fact that they are not adapted to human parasitism, manifesting themselves as *tinea faciei*, *tinea corporis*, *tinea barbae*, *tinea capitis*, Kerion celsi and *tinea unguium*.^2^, ^8^ It mainly affects children and adolescents through direct contact with domestic animals, but it can also appear in young adults and immunosuppressed patients.

The identification of the fungus is made by morphological characteristics, associated with proteomic and molecular techniques.^2^, ^4^, ^9^ The yellowish color observed in the macromorphological examination of the colony suggests *Microsporum canis*; however, the micromorphology is more suggestive of *T. mentagrophytes*, showing rounded or oval microconidia, grouped or implanted laterally in the hyphae. The laboratory technician suspicion is important for the correct identification of the colony, confirming it through molecular or proteomic characteristics, as in the reported case.^2^, ^10^

The treatment of choice for extensive cases is oral terbinafine. In isolated lesions, topical therapy with azoles, terbinafine, or cyclopiroxolamine is indicated, with excellent response.^2^

The clinician must be aware of new species of fungi as etiological agents of cutaneous mycoses, especially in children, due to the current diversification of domestic animals in contact with humans. The laboratory technician needs to be prepared to suspect and identify new species of fungi as etiological agents of cutaneous mycoses, especially infections caused by *T. benhamiae*, in which the usual morphological identification is not so characteristic. It is up to the veterinarian to advise animal owners about the possibility of the transmission of cutaneous mycoses to those who live with them.

The integration of different specialties and laboratory support is invaluable for a favorable patient outcome.

## Financial support

None declared.

## Authors’ contributions

Cristiana Ludwig Schneider Longo: Design and planning of the study; drafting and editing of the manuscript; collection, analysis, and interpretation of data; intellectual participation in the propaedeutic and/or therapeutic conduct of the studied case; critical review of the literature; approval of the final version of the manuscript.

Flávio Marcondes Hercules: Collection, analysis and interpretation of data; effective participation in research orientation; critical review of the literature; approval of the final version of the manuscript.

Fábio Silva de Azevedo: Collection, analysis and interpretation of data; effective participation in research orientation; critical review of the literature.

Adriana Lúcia Pires Ferreira: Collection, analysis and interpretation of data; effective participation in research orientation, critical review of the literature.

Rosane Orofino-Costa: Design and planning of the study; drafting and editing of the manuscript; collection, analysis and interpretation of data, effective participation in research orientation; critical review of the literature; approval of the final version of the manuscript.

## Conflicts of interest

None declared.
